# State-Transition Modeling of Human–Robot Interaction for Easy Crowdsourced Robot Control

**DOI:** 10.3390/s20226529

**Published:** 2020-11-15

**Authors:** Masaya Iwasaki, Mizuki Ikeda, Tatsuyuki Kawamura, Hideyuki Nakanishi

**Affiliations:** 1 Department of Adaptive Machine Systems, Osaka University, Suita 565-0871, Japan; masaya.iwasaki@ams.eng.osaka-u.ac.jp (M.I.); mizuki.ikeda@ams.eng.osaka-u.ac.jp (M.I.); 2 Kyoto Innovation, Inc., Kyoto 604-8151, Japan; kawamura@kyotoinnovation.jp

**Keywords:** robotic salesperson, field trial, multimodal conversation analysis, situation awareness

## Abstract

Robotic salespeople are often ignored by people due to their weak social presence, and thus have difficulty facilitating sales autonomously. However, for robots that are remotely controlled by humans, there is a need for experienced and trained operators. In this paper, we suggest crowdsourcing to allow general users on the internet to operate a robot remotely and facilitate customers’ purchasing activities while flexibly responding to various situations through a user interface. To implement this system, we examined how our developed remote interface can improve a robot’s social presence while being controlled by a human operator, including first-time users. Therefore, we investigated the typical flow of a customer–robot interaction that was effective for sales promotion, and modeled it as a state transition with automatic functions by accessing the robot’s sensor information. Furthermore, we created a user interface based on the model and examined whether it was effective in a real environment. Finally, we conducted experiments to examine whether the user interface could be operated by an amateur user and enhance the robot’s social presence. The results revealed that our model was able to improve the robot’s social presence and facilitate customers’ purchasing activity even when the operator was a first-time user.

## 1. Introduction

Service robots, such as reception robots, clerk robots, and nursing care robots, have become increasingly widespread, and research has been conducted on museum guide robots [1,2], robots for education [3], and service robots in hotels [4,5]. In this study, of the various communication robots, we focus on robotic salespeople in shops.

Overseas travel has increased in recent years, and there are many shops where the clerks cannot speak multiple languages and find it difficult to communicate using a foreign language. Robotic salespeople can be helpful in such shops by attracting foreign customers and describing products in multiple languages. In addition, robotic salespeople can provide services according to the customers’ purchase frequency and product preferences based on their purchase history. Moreover, customers are less likely to hesitate to continue asking questions and can be more carefree in their behavior with robotic salespeople than with human salespeople. We believe robots have some unique advantages that salespeople do not, and the opposite can be said as well. Therefore, in this study, we focus on not the replacement of human salespeople as robots but the integration of robots and human salespeople as one sales team.

Recently, many studies on robotic salespeople have been conducted. There was a similar study using a robotic salesperson in an actual store, where a semi-autonomous robot distributed coupons in shopping malls [6]. However, the robot did not directly introduce the store’s products to the customers. Similarly, current autonomous robotic salespeople can only perform predetermined actions and cannot handle unexpected situations in a timely and appropriate manner. This problem can be solved if the robots are remotely controlled by humans. However, using these robots may be infeasible due to the need for operators with specialized knowledge and the labor costs involved in hiring them. We believe that general users on the internet can operate robotic salespeople remotely and provide flexible customer service in various situations. However, the robots’ remarks and suggestions can be easily ignored by people due to its weak social presence, which is difficult for first-time users to improve. Thus, the goal of this study is to develop a user interface for general users that allows them to remotely control robotic salespeople installed in an actual shop and to enhance their social presence enough for customers to accept their suggestions.

In this study, we examined how our developed remote interface can improve a robot’s social presence while being controlled by a human operator, including first-time users. We conducted an experiment in an actual shop to investigate the typical flow of a customer–robot interaction that is effective for sales promotion, and created a state-transition model of the interaction. In addition, we developed a user interface for the robotic salesperson that could be controlled based on that model. Some of the robot’s behavior was automated by accessing real-time sensor information, which assisted the user’s operation and made the operation easier. Finally, we investigated whether the user interface was effective for sales promotion in a real-world environment.

## 2. Related Works

### 2.1. Service Robot

A number of studies have considered service robots. One study examined the installation of a robot in an information center to attract customers; however, the size of the dataset was insufficient [7]. Moreover, personalizing human–robot interaction contents can enhance the social interactions [8]. In other research, it was demonstrated that when a robotic salesperson attempted to start a conversation with customers in a real store, the robot could succeed in interactive conversation by making a short speech that was easy to respond to [9], and the robot’s presence was strengthened by indicating that it understood the state of the customer [10]. In the present study, we examined which series of actions are useful when a robot provides services to customers.

### 2.2. Remote Control of a Robot

Research has been conducted to create a dialog system for a robot that combines automatic response and remote control [1], in which the robot and remote operator provide customer service according to their own strengths using a remote control system. However, the limitation of this approach is that the remote operator must have specialized knowledge about the shop. In addition, the study did not investigate whether the robot’s automatic responses were appropriate for customer service. Therefore, in this study, we developed a user interface that does not require the operator to have specialized knowledge about the shop when operating the robot.

It has also been demonstrated that it is effective to provide a robot with many autonomous functions as a method for reducing the workload of the user in remotely controlling the robot [11,12]. In this study, we developed a user interface in which autonomous operation and remote control of a robot are mixed. This interface can be easily operated by general users who do not have specialized knowledge, and can reduce the workload of the operator.

## 3. Modeling of the State-Transition in a Customer–Robot Interaction

In this study, we examined natural interactions that cannot be observed in a laboratory experiment, and conducted an experiment to install a robotic salesperson in a real shop. In addition to obtaining verbal information from the conversation between the customers and robot, we performed a multimodal analysis of nonverbal information, and investigated the robot’s behavior to identify aspects that can help promote the purchasing activities of customers.

### 3.1. State-Transition in Customer–Robot Interaction

To allow a robotic salesperson to be operated by general users, it is desirable for the user interface to only allow users to use predetermined rules, as some users have no experience in robot operation. To this end, we performed a multimodal conversation analysis using verbal and nonverbal information obtained from the experiment, in which the robot interacted with customers in the shop, to model the state transition of the interaction between customers and robot. We considered that three major behaviors are required when a robot provides services to customers in a real environment:

1. Draw the attention of customers,
2. Start a conversation with the customers, and
3. Continue the conversation and arouse customers’ interest in the product.

First, drawing the attention of customers and stimulating their interest in the robot will lead them to listen to the robot’s remarks. Then, the robot can start a conversation with the customers and naturally describe a product during the conversation. Therefore, based on the finding that a robot should make short and easy-to-answer statements at the start of an interaction [9], in our study, the robot first performed a simple greeting to draw the attention of the customers, and then proceeded to have a conversation and describe the product. In this way, the robot behavior was designed. There were three possible states of customers in the shop: high interest in products, high interest in the robot, and low interest in the products and robot. However, it was unknown which type of actions the robot should take (and with which timing and order) in a real environment in which the state of customers can constantly change. Therefore, our experiment was conducted in a real shop, and the interaction between the customer and robot was modeled as a state-transition model.

### 3.2. Experimental Method

#### 3.2.1. Experimental Environment

**Figure 1:**
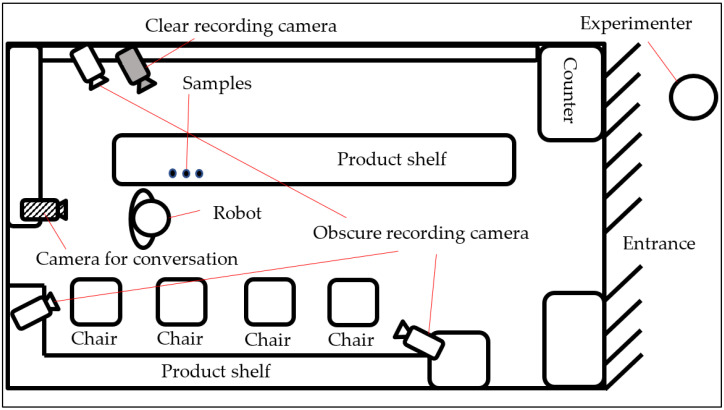
Top-down view of the shop, Dintora, displaying all camera locations.

**Figure 2:**
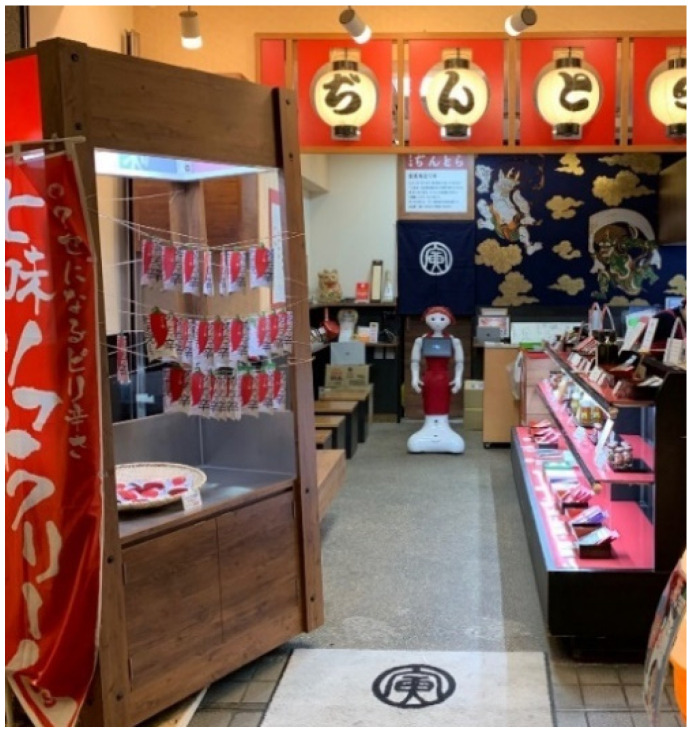
Interior of the shop as viewed from the entrance.

In this study, an experiment was conducted at a shichimi shop called Dintora, a specialty shop located on a shopping street in Kyoto, Japan. Shichimi is a Japanese spice mixture and it is blended by seven different spices. Figure 1 presents a top-down view of the shop, while Figure 2 displays the interior of the shop as viewed from the entrance. In this experiment, as illustrated in Figure 1, three cameras were set up in front of, behind, and to the left of the robot. The cameras were configured so that customers’ faces would not be visible, thereby protecting their privacy. In addition, a consent form was displayed when a customer approached the robot. When the customer has provided consent, a video recognizing the customer’s face will be recorded by the camera focused on the side of the robot.

#### 3.2.2. Robot

Pepper, a product of SoftBank Robotics, was adopted as the robotic salesperson in this experiment due to its appropriate size. Pepper could be seen from the outside of the shop and did not require a large space. Moreover, Pepper had a high safety level and was equipped with internal sensors, such as an infrared sensor and sonar sensor to avoid accidents in the shop. These sensors could also be used to collect nonverbal data from customers, including their facial expressions and physical distance from the robot.

#### 3.2.3. User Interface for Operating the Robot

**Figure 3:**
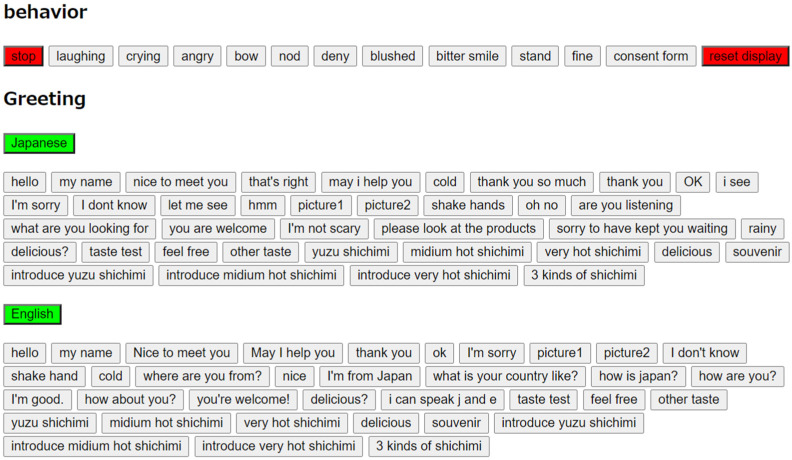
User interface for robot operation.

In this experiment, we controlled Pepper through the Wizard of Oz method, which is an effective simulation method in the development of communication systems. A person pretending to be a system engages in conversation with a user, and during this time, the user believes that he or she is talking to the system. Although the Wizard of Oz method was used in the field experiment, users do not particularly mind whether the robot is operated. It has been demonstrated that the results are not affected by users’ perception of whether the robot is being operated or not [13]. In this experiment, a note posted on the body of the robot informed the customers that part of the robot’s motions would be remotely controlled; however, many customers seemed to not be aware of this. We developed an application that allowed us to control Pepper remotely using a smartphone. Pepper had various functions, such as greeting customers, introducing products, requesting a handshake, offering to try a sample, and calling for a salesperson. The operating system (OS) of Pepper was NAOqi OS and contained application programming interface (API) modules for acquiring and controlling the status of the devices and memory installed on Pepper. Pepper could be operated using a browser from a personal computer or a smartphone connected to the same network as Pepper using the LibQi JavaScript SDK library provided by SoftBank Robotics in Hypertext Markup Language (HTML) format. During the experiment, including all possible actions and voice content using a browser while operating Pepper would require a long time and conversation would thus not be smooth. Therefore, we developed a series of behaviors that could be used for customer service in the shop using Choregraphe, a software used to develop Pepper’s applications, and installed it on Pepper in advance. This allowed us to use the actions and voice content pre-installed on Pepper by the push of a button. With the Wizard of Oz method, it took time for the operator to determine the robot’s next action, but it was not long enough to be a significant problem when the robot is interacting with the visitors. In this experiment, the user interface for the robot’s operation illustrated in Figure 3 was used. In this user interface, the buttons for the robot’s customer service behavior were arranged on one screen so that the operator could freely select them. This made it possible for the robot to act flexibly according to the behavior of the customers. In addition, we were able to investigate which behavior of the robot was effective for selling products depending on the state of the customers.

#### 3.2.4. Acquisition of the Remote Video

In this experiment, remote control was performed using a first-person viewpoint camera mounted on the head of Pepper and a third-person viewpoint camera installed in the shop. Images from these cameras were used after being obscured for privacy protection. The first-person viewpoint camera allowed the operator to see the direction of the customers’ line of sight, while the third-person viewpoint camera allowed the operator to observe both the robot and customers. In addition, sensor information, such as the customers’ gender, age, and gaze direction, was also displayed in the first-person viewpoint image.

#### 3.2.5. Ethical Issues

During the experiment, we displayed a consent form that customers could answer by touching a tablet mounted on Pepper. Only when consent was given, we obtained clearer footage in which the customer’s face could be recognized. In addition, an outline of the experiment, the name of the person leading the experiment, and a contact number were posted on the body of Pepper. Handouts with the same content were left in a tray and placed on the body of Pepper so that customers could take them home. In this experiment, to ensure safety, the moving function of the robot was not used. In case of an emergency, the experimental staff were located nearby so that they could respond immediately. This experiment was approved by the Research Ethics Committee of Osaka University.

### 3.3. Results and Discussion

The experiment was conducted for a total of 20 days in 2017 (November 27; December 2, 4, 5), 2018 (April 1, 2, 16; September 10, 11, 20, 21; December 3, 5, 6, 10, 11), and 2019 (April 17, 20, 22, 25), and provided service to 330 groups of customers.

#### 3.3.1. Starting a Conversation with a Customer

According to previous studies, some researches have been conducted with robots to estimate the internal state of humans [14,15] and to obtain information on conversation partners before a robot starts a conversation [16]. Allowing a robot to read social cues is one of the indispensable means for communicating with humans. There is also a study demonstrating that a robot’s ability to recognize and respond to human behavior is important for the robot to successfully engage with people and persuade them to nod and reply to its comments [10]. Based on this finding, we developed an attention estimation model (AEM) based on data of customers’ nonverbal cues in the field experiment, and examined whether the model was effective in a real-world environment. This field experiment was divided into two steps: (**1**) training the AEM and (**2**) verifying the effectiveness of the AEM in a real-world environment. We used the data of the first step of the field experiment in 2017 for 10 days, and performed the second step of the field experiment in 2018 for 9 days.

According to Poggi [17], engagement is defined as the value that a participant in an interaction attributes to the goal of being together with other participants and continuing the interaction. In this study, we defined customers’ engagement as the probability that they responded to Pepper, the robotic salesperson. According to the following previous studies, we determined that one of the most common factors for estimating people’s engagement is eye contact, which is a strong sign of paying attention to a conversation partner [18,19]. Moreover, eye contact makes it possible to see facial expressions, such as smiles and frowns. However, reactions to the robot, such as nodding and posture, can also provide vital information [19,20]. In addition, the physical distance between the robot and customers is another important cue [21].

From the above, we selected the following five nonverbal cues: (**1**) eye contact, (**2**) duration of eye contact, (**3**) distance, (**4**) approaching, and (**5**) laughing. These could be detected by Pepper’s sensors to calculate the degree of engagement. In this study, verbal cues were not used because there was a large amount of noise in the shop and the robot could not accurately detect verbal cues. We collected data from the video of the first step of the field experiment, in which 86 groups had conversations with Pepper. However, in 11 of the 86 customer groups, the conversations were started by the customers. Therefore, we excluded these 11 groups from the data because their interactions were not consistent with the situations we aimed to investigate. If there were several customers in one group, we focused on the customer closest to the robot. Nonverbal cues from the time at which the customers entered the shop to the robot’s first spoken words were defined as the following variables:

Y: Engagement, binary data that defined whether the customer responded to the robot’s speech (1 if yes, 0 if no).
X1: Eye contact, binary data that defined whether the customer looked at the robot when the robot greeted him or her (1 if yes, 0 if no).
X2: Duration of eye contact, linear data that defined the length of eye contact measured in seconds. This was the period between the customers’ entrance into the shop and the robot’s first greeting.
X3: Distance, linear data that defined the distance between the robot and the customer measured in meters during the robot’s greetings.
X4: Approaching, binary data that defined whether the customer approached the robot when it greeted him or her (1 if yes, 0 if no).
X5: Laughing, binary data that defined whether the customer laughed when the robot greeted him or her (1 if yes, 0 if no).

In this study, inter-rater reliability was assessed for 40 groups of customers (approximately 25% of the entire customer data). The micro-behaviors of the customers were scored by two coders: one who scored the entire customer data, an another who scored 40 groups of customers. The inter-rater reliability results were as follows: global inter-rater reliability (kappa = 0.76), engagement (kappa = 0.79), eye contact (kappa = 0.75), duration of eye contact (kappa = 0.77), approaching (kappa = 0.83), distance (kappa = 0.71), and laughing (kappa = 0.69).

To estimate the engagement of customers, we adopted logistic regression, a simple machine learning model for solving binary classification problems. The results of the training are presented in Table 1. The table indicates that eye contact, duration of eye contact, approaching, and laughing all had a positive correlation with engagement, whereas distance had a negative correlation with engagement. In addition, the bias was −0.20. In this study, we conducted 5-fold cross validation to validate the attention estimation model in our datasets. We achieved 88.9% accuracy, 87.1% precision, and 90.7% recall, which we considered sufficient to estimate the engagement of customers.

**Figure 4:**
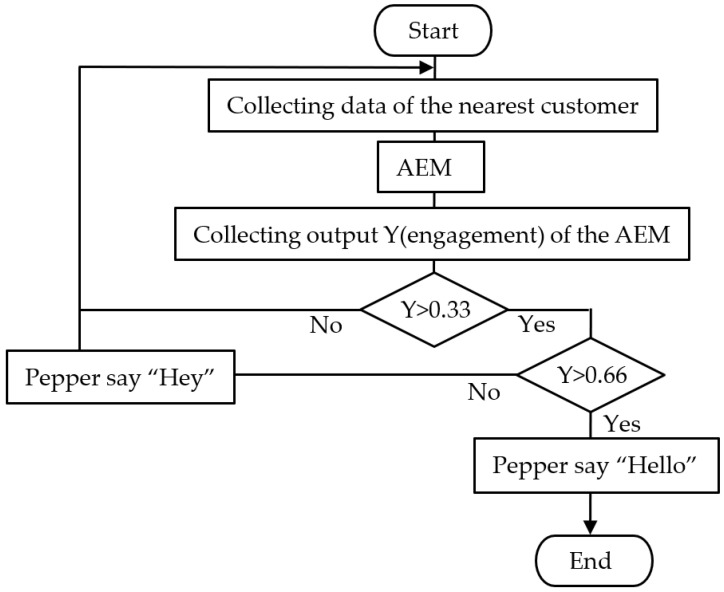
Automatic greeting mode in the robot

Before the second step of the field experiment, we installed an automatic greeting mode based on the AEM in the robot, which is illustrated in Figure 4. The customers’ faces were captured by the camera on Pepper’s head to judge whether the customers had eye contact with Pepper and detect their facial expressions. In addition, we determined whether the customers approached Pepper, measuring the distance by laser radar on Pepper’s feet. The sensors could only detect customers approximately 3 m away from Pepper.

In Figure 4, the output of the AEM was a probability from 0 to 1, and we designed two thresholds to divide the engagement levels of the customers into three categories. If the engagement was lower than 0.33, we programmed Pepper to do nothing because the customers were not interested in the robot at all. If the engagement was between 0.33 and 0.66, we programmed Pepper to say “Hey” because spoken words can attract customers’ attention without the customers being forced to reply. In addition, if the customers’ engagement rose above 0.66, Pepper said “Hello” or asked customers to shake hands, as the customers’ engagement was sufficiently high to lead the customers to respond to the robot’s statements.

**Figure 5:**
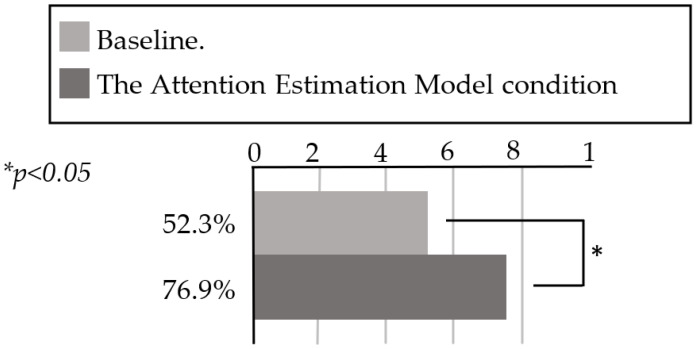
Groups of customers who responded to the robot after it greeted them under two conditions.

In this experiment, we compared two groups of customers: those who responded to Pepper after it greeted them automatically based on the AEM, and those who responded when an experimenter controlled Pepper remotely. The results of the second step of the field experiment are presented in Figure 5. The baseline represents the condition in which the experimenters controlled Pepper remotely (only when the customers approached the robot within 3 m, as this was the distance from the robot to the entrance of the shop). During the experiments, we alternately performed the baseline and automatic greeting mode condition every 30 min. In the AEM condition illustrated in Figure 5, 30 out of 39 customer groups responded to the robot with a proportion of 76.9%. In the baseline condition, 22 out of 42 customer groups responded to the robot with a proportion of 52.3%. We thus determined that the proportion of customers’ responses in the AEM condition was higher than that in the baseline condition. In addition, we performed a chi-squared test to validate our data. The results indicated that there was a significant difference between the two conditions (*X*2 = 5.29, *p* = 0.021 < 0.05). In addition, during the first step of the field experiment, 75 out of 153 customer groups responded to the robot’s remarks with a proportion of 49.0%. Therefore, the automatic greeting mode led to a higher proportion of customer responses than the baseline mode. To investigate the cause of these results, we analyzed the interaction of the following groups.

A conversation with a customer group in which the robot was remotely controlled is presented in Table 2. This transcript indicates that when the robot said “Hello,” although the customer (C1) approached the robot, she did not look at the robot (lines 4 and 5). Thereafter, the robot was ignored. We considered that during the remote control experiments, it was difficult for the controller to determine what the customers were looking at.

A conversation with a customer group in which the robot was set up in the automatic greeting mode is presented in Table 3. It can be seen that when the robot said “Hey,” the customer (C1) turned her head and looked at the robot (lines 3 and 4). Thereafter, the robot perceived the eye contact of C1 and said “Hello” (line 5) rapidly, and C1 then responded to the robot and a conversation was initiated (line 6). The automatic greeting mode appeared to perceive people’s states more precisely, allowing the robot to make decisions more quickly. Therefore, when the robot greeted customers based on the AEM, customers were likely to feel that the robot could understand their degree of attention to the robot. The customers may have then felt a sense of guilt if they ignored the robot. Consequently, they may have been more willing to respond to the robot [10]. Thus, an automatic greeting based on the AEM was effective in a real-world environment and was able to strengthen the robot’s social presence. The robot’s greeting based on the AEM led customers to believe that the robot could understand their behaviors due to the robot’s ability to turn its head toward the customers or make eye contact with them. Thus, creating the impression that a robot can detect people’s behaviors can lead to a higher probability of customers responding to the robot’s remarks [22].

However, there were some customers whose engagement value did not increase enough for the robot to greet them. If the automatic greeting mode is used for such customers, the robot must take action to attract the customers’ attention. For example, rhythmic speech can be novel and interesting. Therefore, in this experiment, rap singing was adopted to further attract customers’ attention. Rap singing was performed by the robot when the value of the target customer’s engagement with the robot was not high enough for the robot to greet him or her automatically and when the customer was likely to leave the shop if the robot did nothing. Thus, to identify these scenarios, we counted the number of groups that the robot greeted every 10 s after entering the shop and the number of groups that left the shop before the robot’s automatic greeting function was activated, and examined their relation.

**Figure 6:**
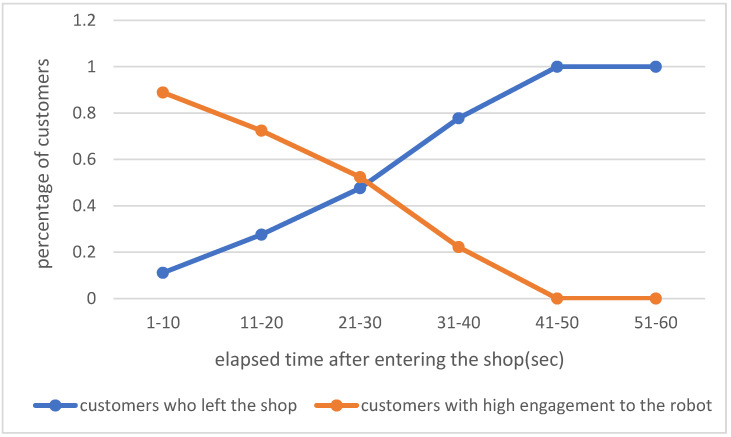
Changes over time in the percentage of customers greeted by robots and departing customers when using the automatic greeting mode (high engagement: value more than 0.6).

The results presented in Figure 6 indicate that 73 customers visited during using the automatic greeting mode, with 42 groups greeted by the robot and 31 groups leaving the shop before the robot could greet them. The horizontal axis in Figure 6 represents the elapsed time from when the customer entered the shop when the robot greeted him or her and when the customer left the shop, while the vertical axis represents the ratio of the number of customers every 10 s. This figure indicates that after 21 to 30 s passed after entering the shop, the number of groups leaving the shop before the robot could greet them was greater than the number of groups with a high engagement level. This signifies that if the robot did not greet the customers using the automatic greeting mode 21 to 30 s after their entrance into the shop, the customers were likely to leave. Therefore, this was considered the appropriate time to perform rap singing to attract the customers’ attention. If the engagement value was not high enough for the robot to greet the customers even after 25 s (which is between 21 and 30 s), it was deemed that the robot should sing a rap song.

#### 3.3.2. Continuing Conversations with Customers

**Figure 7:**
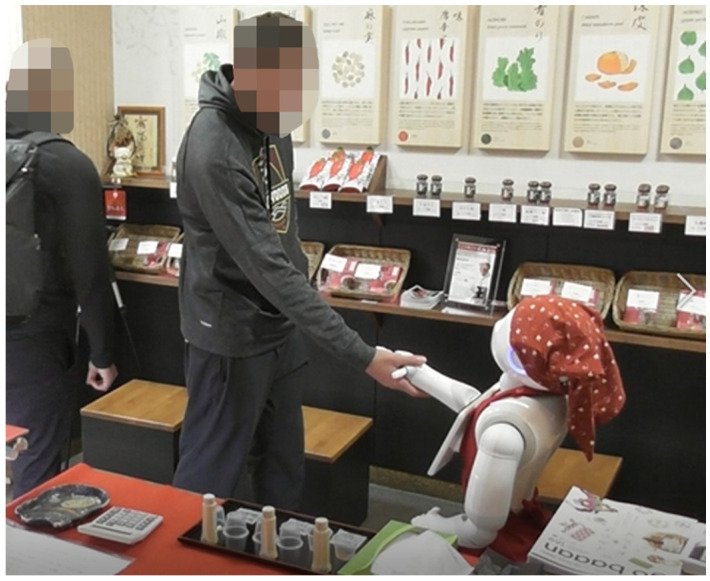
Pepper successfully engaged a customer with a handshake.

For customers who started a conversation with the robot using the automatic greeting mode, the robot had to behave appropriately to continue the conversation. Requesting a handshake was one of the actions the robot performed in this experiment. There has been a previous research on how a robot’s handshake demonstrates human-likeness through the way the robot’s arm moves [23]. When requesting a handshake in this experiment, the robot moved its hand forward, as illustrated in Figure 7, and requested a handshake while saying, “May I shake hands with you?” A handshake is a typical greeting in human-to-human communication; however, with human salespeople, the act of shaking hands with a customer is not very common. Nevertheless, we considered that the robot’s handshake would be effective for drawing customers’ attention. In this experiment, when requesting a handshake for groups of customers after they started conversations with the robot, 117 out of the 128 customer groups (approximately 91% of cases) responded to the handshake. Therefore, handshaking can be considered useful as an action for continuing a conversation with customers.

#### 3.3.3. Shifting Customers’ Attention to Products

**Figure 8:**
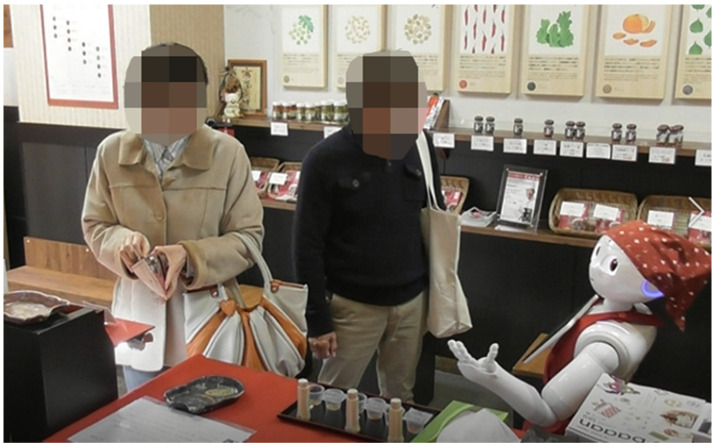
Robot’s movement when suggesting to taste a sample.

We also considered a motion to suggest tasting a sample as a method to shift attention from the robot to the products. Sample tasting has been shown to be effective in promoting sales [24], where the psychological effect called the norm of reciprocity affects the customer, who feels that something must be returned after receiving something from another person. In addition, a study has been conducted in which tasting was performed using a robot in a field experiment [25]. In the study, the authors compared the abilities of a robot and humans to successfully advertise sample tastings to customers in large shopping centers, and demonstrated that the robot was more effective than humans. When the robot offered a sample to the customers, it said “Would you like to try a sample? You can taste here!” and indicated the sample using its hand and eyes, as illustrated in Figure 8.

**Figure 9:**
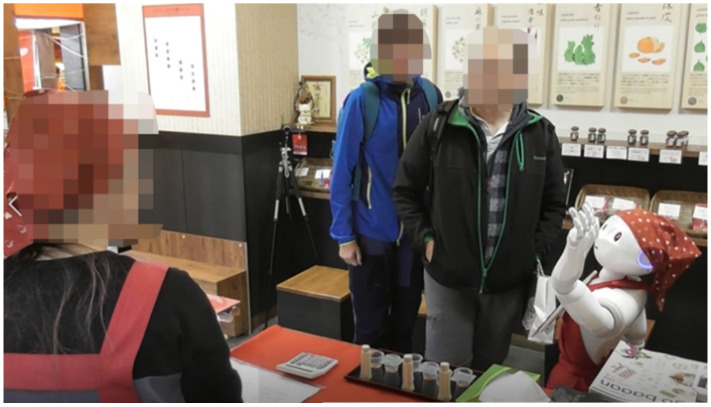
Robot’s movement to ask for a salesperson’s help.

However, even when the robot suggests tasting a sample, the customers do not always respond to the request. Therefore, an aggressive approach, such as handing a sample to customers, can lead customers to behave according to the consistency principle, which is a psychological process in which people tend to accept purchase requests in order to keep their behavior consistent. However, it is not easy for a robot to take something with its mechanical hand and hand it to the customer. The reason is not only that dexterous hand movements are difficult for a robot, but also that customers may not believe that the robot can respond accordingly even if they express a desire to try a sample. In attempting to solve these problems, it is necessary to consider a method of handing the sample directly to the customers. We decided that it would be sufficient to complete the task of having the customers agree to a sample tasting. Therefore, in this experiment, we installed a function for the robot to request a salesperson to give customers a sample for tasting. Specifically, the robot called a salesperson with the motion illustrated in Figure 9 while saying “Excuse me. Please give them a sample.”.

To determine when to activate the motion to suggest tasting a sample and the request for a salesperson’s assistance, we compared a group that agreed to taste a sample and a group that did not agree after the robot’s suggestive motions. Table 4 presents a conversation with a group to which the robot suggested tasting a sample. In this conversation, the robot requested the help of a salesperson to distribute the sample to the customers, and the customers then purchased the product.

In the example displayed in Table 4, when the customer (C1) looked at the robot while talking, the robot suggested tasting a sample by saying “Would you like to try a sample? You can taste here” (line 16), and the customers turned their heads to the sample and replied to the robot (line 18). Seven seconds later, while the customer was looking at the sample, the robot requested help from the salesperson (line 21), and the salesperson passed the sample directly to the customer (line 25). The customer then tasted and purchased the product. In this example, the customers were obviously interested in the robot, as they took pictures of it; however, the robot appeared to successfully promote sales by directing their attention to the products with suggestions to taste a sample.

We also analyzed a conversation with a group that did not taste a sample after the robot’s suggestion. Table 5 presents a conversation with a group that responded to the suggestion to taste a sample but did not taste it even after the robot asked the salesperson for assistance.

In Table 5, the customer (C1) looked at the robot in response to the robot’s suggestion “Would you like to try a sample?” (line 9). When the robot said “You can taste here” (line 10), the customer looked at the sample (line 11). However, during the robot’s description of the sample, the customer turned away from the sample, seeming uninterested (lines 14 and 15). At this time, the robot requested assistance from the salesperson (line 18), but the customer ignored the sample and left the shop. Comparing the results of the conversation in Table 4, in which the robot was able to achieve a sample tasting, we observed that there was a difference in the line of sight of the customers when the robot requested assistance from the salesperson.

Next, Table 6 presents a conversation with a group that did not taste the sample when the robot offered to taste it.

In Table 6, the customer (C1) immediately engaged with the robot and responded to the request for a handshake (line 13). Thereafter, when C1 looked away from the robot (line 19), the robot suggested tasting the sample (lines 20 and 22). It should be noted that C1 responded to the statement of the robot’s suggestion to taste (line 21), indicating that he had heard the robot’s suggestion. However, he did not taste the sample and did not even look at it. In this example, when comparing this conversation with the conversations in Table 4 and Table 5, there was a difference in the line of sight of the customers when the robot suggested tasting the sample. Therefore, this analysis reveals the following two points:

1. If a robot offers a sample while the customer is looking at the robot, the customer often looks at the sample for tasting.
2. If the robot asks the salesperson for assistance while a customer is looking at a sample for tasting, the customer often tastes the sample.

Therefore, the results presented in Table 2 were obtained by examining the line of sight of 16 customer groups at the time of the robot requesting assistance from the salesperson.

**Figure 10:**
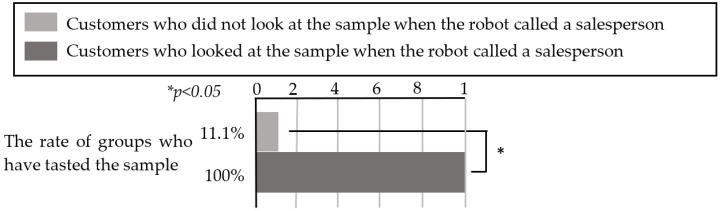
Rate of groups who tasted the sample when the robot called for a salesperson.

According to Table 7, seven groups of customers who looked at the sample before the robot’s request received a sample from the salesperson and tasted it after the robot requested assistance. In contrast, eight groups who did not taste the sample were not looking at the sample before the robot’s request to the salesperson. When Fisher’s exact test [26] was applied to these results, as illustrated in Figure 10, we determined that the groups of customers who were looking at the sample when the robot requested assistance had a significantly higher tasting rate (*p* < 0.05).

Therefore, the results indicate that if the robot requests assistance from the salesperson when customers are looking at the sample and the salesperson gives the sample to the customers, the customers will taste the sample. From these results, it is also possible that if the robot additionally describes the tasting while the customers are looking at the sample (i.e., if they are interested in tasting), the customers may taste the sample. We also determined that the line of sight of the customer is important when the robot requests the salesperson’s assistance. Therefore, we examined the line of sight of 96 groups of customers to whom the robot suggested tasting a sample during the experiment, and Table 8 presents the results.

**Figure 11:**
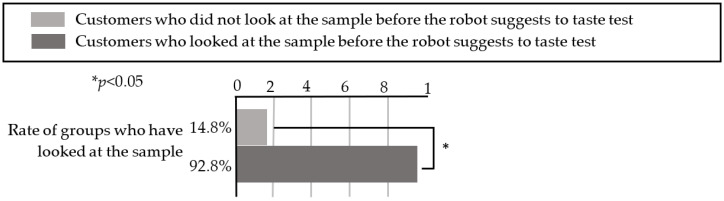
Rate of groups who looked at the sample after the robot suggested a tasting.

According to Table 3, 39 of the 42 customer groups (92.9% of cases) who were looking at the robot during the robot’s suggestion looked at the sample afterwards, while 8 of the 54 customer groups (14.8% of cases) who were not looking at the robot during the suggestion looked at the sample afterwards. A chi-squared test was performed on these results, and the results indicated that the customers who were looking at the robot when the robot suggested tasting a sample were much more likely to look at the sample after the suggestion than customers who were not looking at the robot (see Figure 11). In addition, all 16 groups who tasted a sample were looking at the robot when the robot made the suggestion. Therefore, we determined that it is important for the robot to suggest a tasting while the customers are looking at it to successfully persuade the customers to taste the sample.

#### 3.3.4. Improvement to Robot’s Suggestion to Taste Function

**Figure 12:**
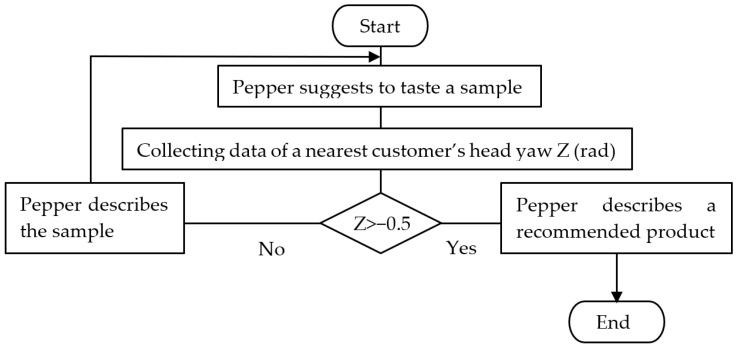
Function of automatic description of products after robot’s suggestion.

The analysis in Section 3.3.3 suggests that it is effective for the robot to perform an action that encourages customers to taste the sample while the customers are looking at the sample. However, if the salesperson is not available, the robot cannot request the salesperson’s assistance. Therefore, we believed that it was necessary to have a function to describe the products that customers could try after the suggestion to taste a sample. We developed a function in which the robot automatically described the products that could be tasted based on the direction of the customer’s gaze after the robot’s suggestion to taste a sample. We used the ALGazeAnalysis API to detect the gaze direction, and acquired the rotation angle around the yaw axis. A positive value was obtained when the target individual looked to the right of the robot. Here, it was not necessary to accurately determine whether the customers looked at the sample. If the customers turned their heads to the left approximately 30 degrees (−0.5 [rad]) or more from the robot, the robot determined that the customer was looking at the sample. The program of the function to automatically describe the products that the customers could taste after the robot’s suggestion is presented in Figure 12.

### 3.4. Development of a Flowchart for the Robot’s Customer Service

The experimental results indicate that the state-transition model of the interaction between the customers and robotic salesperson can be roughly divided into three parts: a greeting part, a conversation continuation part, and a product description part. The state of interaction transits based on the behavior of the customers, from a customer’s entrance to the robot’s attempt to introduce the product to the customer through a sample tasting. The final state of the flowchart is a state in which the customers are tasting the sample and a state in which they are not tasting the sample. In the case of the former, the robot introduces the recipe for shichimi, and in the case of the latter, the robot recommends other products, as the customers may be interested in something other than products that can be tasted. The customer service flow ends when either of these is completed.

**Figure 13:**
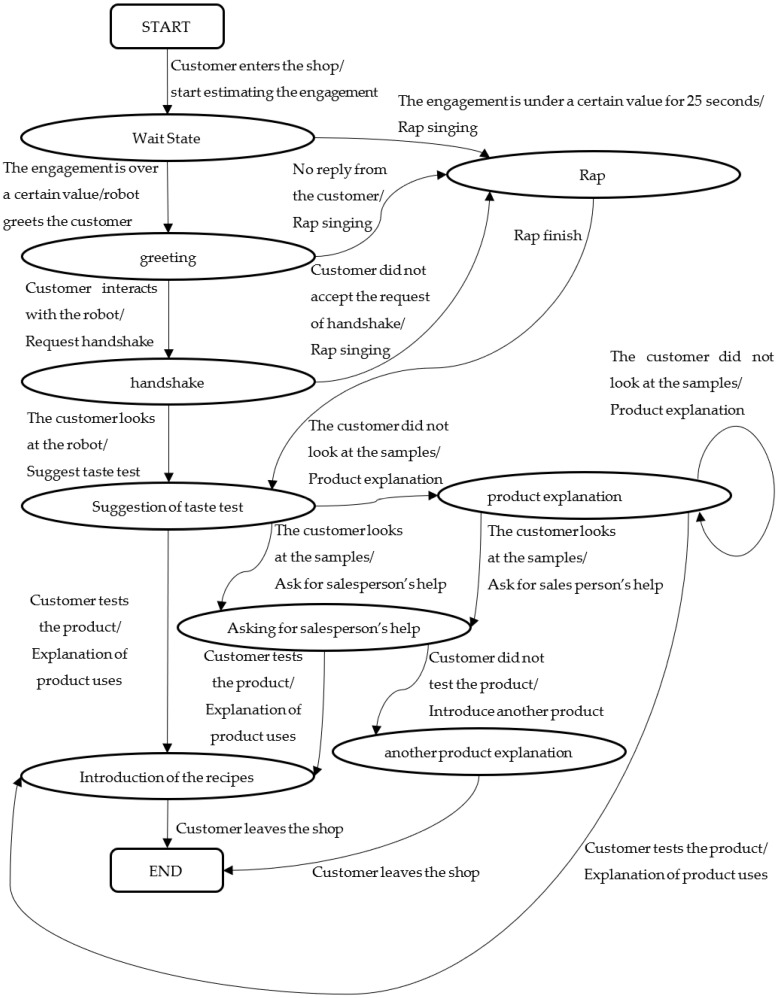
State transition of the interaction between the customers and robot.

**Figure 14:**
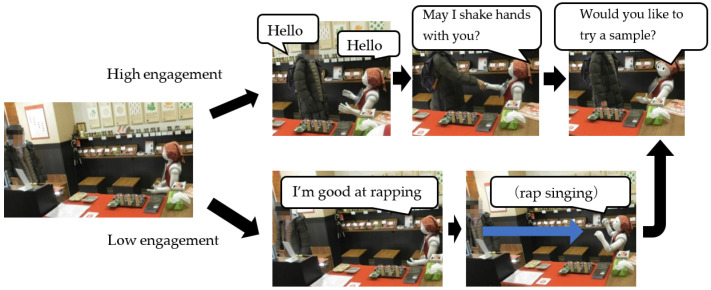
Part of the state transition based on the interaction between the customers and robot.

Figure 13 illustrates the state transition of the aforementioned interaction between the customers and robot. The arrows in this figure represent state transitions, and the label for each arrow is written as “trigger (/robot action).” The trigger represents the factor that causes the transition, and the state changes by the robot’s action. Figure 14 presents part of such a transition of the interaction between a customer and robot in an actual shop. In this user interface, when an operator presses a button according to the condition written on the screen, the contents of the operation of the action that the robot should perform next are automatically presented. The displayed conditions are in effect the customer’s behavior that corresponds to the robot actions. Thereafter, the operator repeatedly evaluates the condition and selects the button for operating the robot according to the flow of effective customer service for sales.

## 4. Verification of Effectiveness of the State-Transition Model in a Shop

In the experiment described in this section, an experimenter controlled Pepper through a user interface based on the state-transition model of customer–robot interaction developed in Section 3, and we investigated the effectiveness of the interaction state-transition model in an actual shop.

### 4.1. Experimental Method

**Figure 15:**
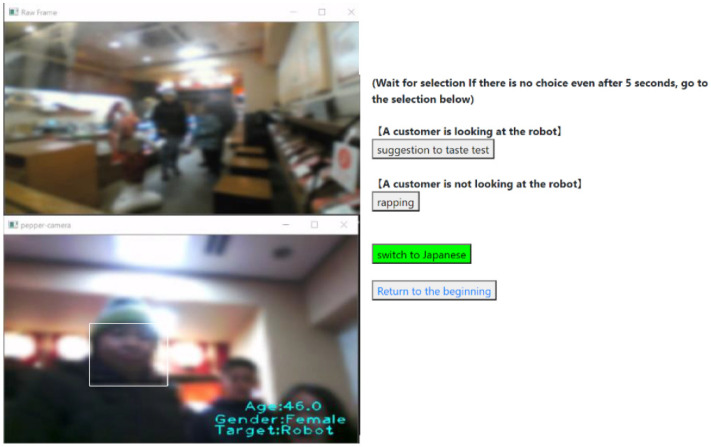
User interface for the robot’s operation.

The experimental environment was the same as that described in Section 3, and the experimenter operated the robot at a remote location using the user interface based on the state-transition model developed in Section 3. Figure 15 presents the user interface for the robot’s operation used in the experiment. In this figure, the third-person viewpoint image is displayed in the upper left of the screen, while the first-person viewpoint image of the robot is displayed in the lower left of the screen. These images were obscured and displayed with blurring through image processing to protect the customers’ privacy. Three pieces of information acquired by the NAOqi API consisted of the customer’s age (0–75), gender (Male, Female), and gaze direction, and were displayed on the first-person viewpoint image. The gaze direction of the customer was classified as one of three cases: (**1**) looking at the robot (Robot), (**2**) looking at the samples for tasting (Sample), or (**3**) looking at other places (Another).

### 4.2. Experimental Results

The experiment was conducted for 10 days in 2019 on October 7, 8, 19, 20, 20, 21, and 22, and on November 20, 21, 25, and 26. In this experiment, 177 groups of customers visited the shop, and 57 groups spoke with the robot.

**Figure 16:**
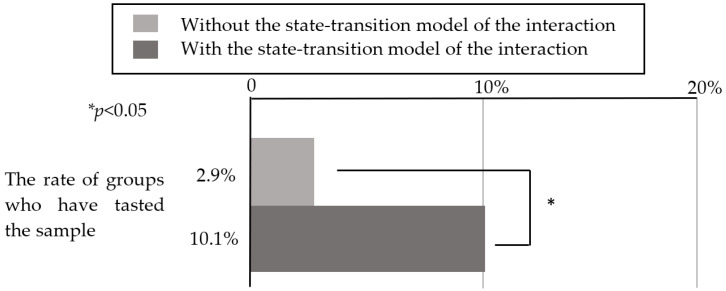
Percentage of customers who tasted a sample.

**Figure 17:**
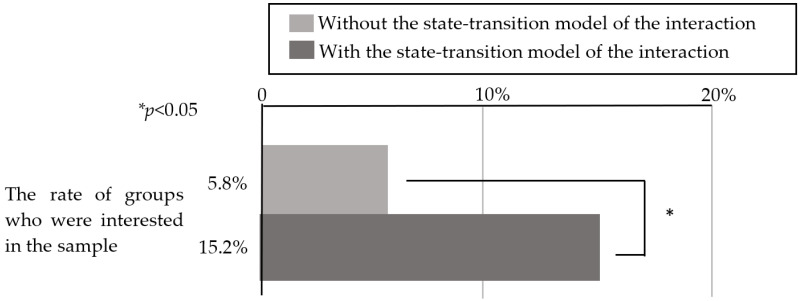
Percentage of customers who were interested in the sample.

To determine whether the user interface enhanced the robot’s social presence enough for the customers to accept its suggestions, we compared the data from this experiment (with the state-transition model) with data from the experiment from Section 3 (without the state-transition model), and investigated whether the customers responded to the robot’s suggestions to taste a sample. Here, to minimize factors other than whether the state-transition model was used, we compared using only the experimental data from December 2018 to April 2019 in Section 3, which used the same functions installed in the robot as those in this experiment. The robot’s location, appearance, and the sample’s location were the same in both experiments. A total of 18 of the 177 customer groups (10.1% of cases) agreed to taste the sample in this experiment, while 5 of the 172 customer groups (2.9% of cases) agreed to taste the sample in the experiment without the state-transition model. Here, the population parameter was the total number of customers the robot interacted with regardless of whether a conversation occurred. The chi-squared test was performed on these data, and the results are presented in Figure 16. We determined that the tasting rate of customers was significantly higher in the operation based on the state-transition model (*x*^2^ = 7.47, *p* = 0.006 <0.05). In addition, the percentage of customers who tasted a sample in this experiment was approximately 10%. However, 27 of the 177 groups were interested in the product, including customers who tasted, picked up the samples, and smelled the product. Similarly, upon comparing this experiment with the experiment in Section 3, we observed a significant difference, as illustrated in Figure 17 *x*^2^ = 8.20, *p* = 0.004 < 0.05). Therefore, the state-transition model used in this experiment was considered effective in a real environment.

### 4.3. Discussion

#### 4.3.1. Effects of Modeling Interaction

**Figure 18:**
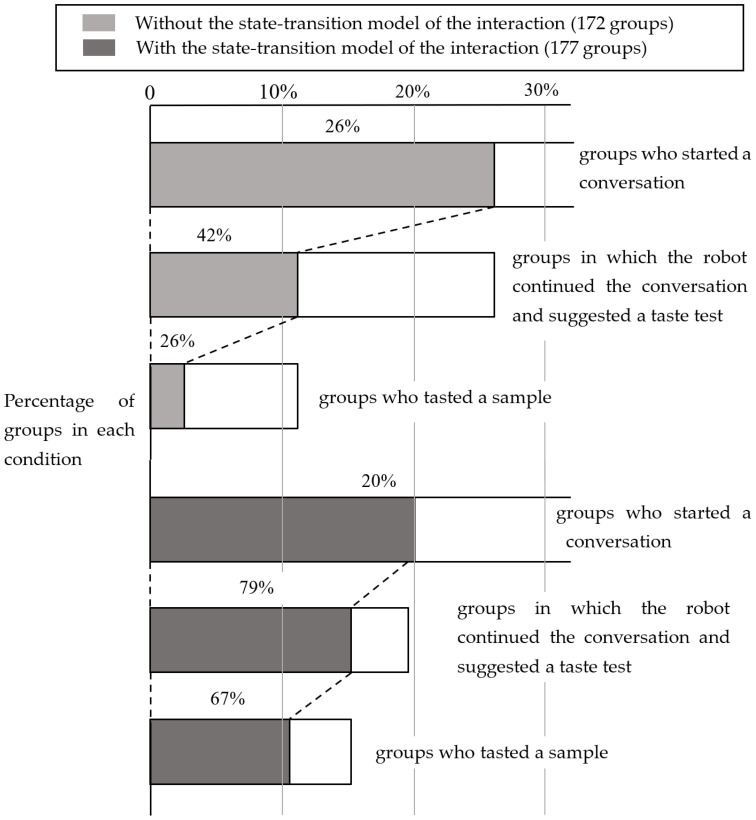
Percentage of customers during each stage of customer service under two conditions.

Figure 16 indicates that by using the user interface for the robot’s operation based on the state-transition model of the interaction between the customers and robot, the proportion of customers who accepted the robot’s suggestion to taste a sample increased. The reason for this is described below. This model roughly consists of three interaction states: a greeting part, conversation continuation part, and product description part, including the suggestion to taste a sample. To determine which part of the interaction model was effective, we set up three stages of customer service: one in which the robot was able to start a conversation, one in which the robot continued the conversation (suggested a sample tasting), and one in which the robot succeeded in leading the customers to taste a sample. For each condition, we investigated the stage that the customers could reach, and the results are presented in Figure 18.

First, the number of customer groups that the robot started a conversation with was 45 out of 172 customer groups (26.1% of cases) without the state-transition model, and 34 out of 172 customer groups (19.7% of cases) with the state-transition model. It should be noted that of the groups that the robot could continue a conversation with and suggest tasting a sample, 27 out of the 34 customer groups (79.4% of cases) started a conversation with the robot with the state-transition model, while only 19 out of the 45 customer groups (42.2% of cases) started a conversation with the robot without the state-transition model. Therefore, it appeared to be more difficult for the robot controlled without the model to continue a conversation, thus contributing to the difference in the final tasting rate. In addition, the proportion of groups that tasted a sample after the suggestion was 26% without the model and 67% with the model. A possible reason for this result is the difference in the timing at which the robot suggested tasting a sample. With the state-transition model of interaction, the robot suggested tasting a sample when the customers were looking at the robot. In contrast, without the model, the robot suggested tasting a sample regardless of whether the customers were looking at the robot. Next, we consider the actual conversation that occurred in these experiments. Table 9 presents a conversation with a group that tasted a sample with the state-transition model.

In Table 9, the robot started a conversation with the customers, continued the conversation by shaking hands, and then suggested tasting a sample while the customers were looking at the robot. The customer then turned to the sample (line 10). Thereafter, one of the customers (C1) picked up the sample and tasted it. As a result that these customers greeted the robot immediately after entering the shop, they likely entered the shop with a high degree of interest in the robot. However, when the robot suggested tasting a sample, the attention drawn by the robot was shifted to the sample, and the robot led the customer to taste the sample. Thus, the model used in this experiment can be appropriately applied to customer service in an actual shop. In contrast, Table 10 presents a conversation in which the robot operating without the model started a conversation with the customers but could not continue the conversation and suggest tasting a sample.

In Table 7, the robot started a conversation and the customers asked the robot a question. Therefore, it was assumed that the customers had some interest in the robot. However, the robot’s reply was not appropriate, and the customers lost interest and left the robot at the stage of continuing the conversation. There was also a time at which the customers looked at the robot (lines 10–13); however, the customers left the shop without being suggested tasting a sample. As illustrated in this example, a robot operating without the model may not be able to properly introduce the product due to a long conversation that is unrelated to the product. Thus, the state-transition model of interaction appears to be effective in preventing a situation in which the customer leaves the robot before being suggested tasting a sample. Next, Table 11 presents a conversation with the customers in which the customers started the conversation and the robot suggested tasting a sample in the operation without the model, but the customers did not taste the sample.

In Table 8, the robot was able to start a conversation, and while trying to continue the conversation, it took some time for the robot to reply (lines 6–7, 15–17), even though the conversation was natural. However, as in the case of Table 7, the robot suggested tasting a sample when the customers were not looking at the robot. Instead, the robot should have suggested tasting a sample while the customers were looking at the robot (line 16). In the case of operation based on the state-transition model, we believe that the number of customers who tried to taste a sample increased because the robot smoothly led the conversation from the beginning and suggested tasting a sample with better timing, as in Table 6.

**Figure 19:**
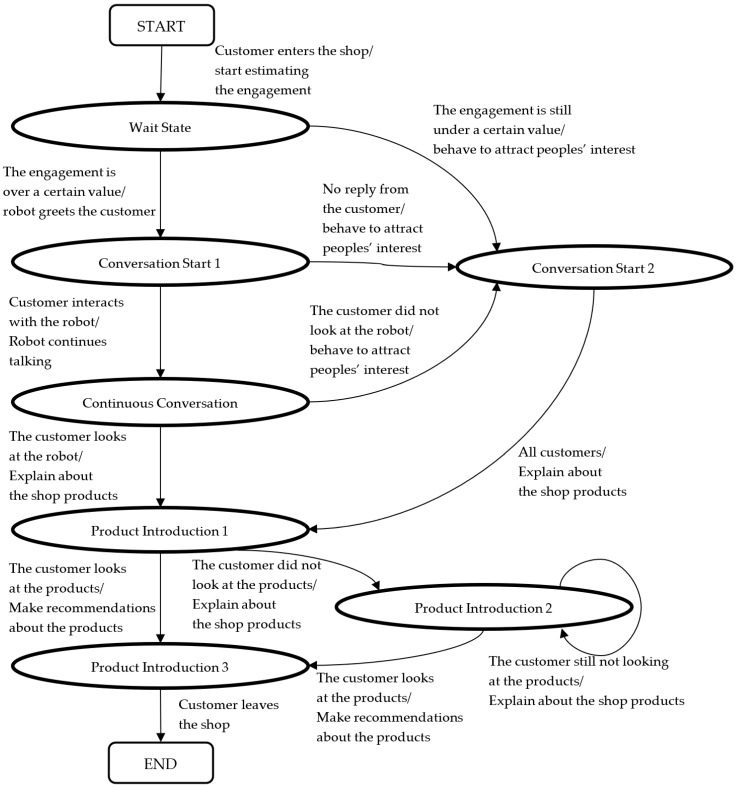
Abstracted state-transition model of interaction.

From the above, we concluded that the state-transition model of the interaction between the robot and customers is useful to sufficiently improve the robot’s social presence for customers to accept its suggestions in the real environment. The state-transition model in this study was designed to function in a specific shop and environment; however, we believe that it can be applied to other environments by abstracting the model. Figure 19 presents such an abstracted state transition. By designing individual behaviors and functions of the robot based on Figure 19 according to the environment and purpose of the robot, regardless of whether the robot is autonomous or remotely controlled, we believe that the robot’s performance can be improved.

#### 4.3.2. Effect of Improved User Interface for Robot Operation

The user interface for the robot’s operation in this experiment consisted of simple actions that customers were likely to perform in the shop. Therefore, as long as the state in the actual shop changed according to our predetermined transition rules, miscommunication between the robot and customers could be avoided and the robot would be more capable of performing actions suiting the situation. However, if a customer performed many unexpected actions, miscommunication could occur and the robot would potentially be unable to properly provide service to the customers. Therefore, we counted the cases in which the customers’ interest in the robot was lost due to the robot’s inability to respond to their actions and statements that did not correspond to the model of the interaction during the conversation. In this experiment, there was only one such case among the 57 groups who spoke with the robot. The conversation with the group is presented in Table 12.

In Table 12, the customers greeted the robot positively, asked the robot to shake hands often, and said “I like you.” Therefore, we considered that these customers were not interested in the product at all and merely wanted to have a conversation with the robot. We thus determined that there are cases in which this interaction model is not effective for customers who have no interest in the product. However, this was a single case, and we believe that most customers should be at least somewhat interested in the products in the shop. Therefore, in this experiment, we considered that the robot could describe the product without much miscommunication. In addition, in this user interface, it is necessary for the operator to evaluate the reaction of the customers from the robot’s previous action based on the camera image, and the operator can simply press a button according to the situation for the robot to perform the next action. However, as can be seen from the case presented in Table 12, the operator could easily judge the condition during the operation because the robot acted promptly after the customer’s remark.

In contrast, in the experiment described in Section 3, the operator selected a behavior that seemed appropriate for the situation from the robot’s many possible behaviors and performed customer service accordingly. Although it may be possible to perform customer service more flexibly than the operation using the user interface described in this section, it takes time to select buttons, and operation errors are more likely to occur due to the degree of freedom in operation. Table 13 presents a conversation with a group in which an operation error was observed.

In Table 13, the robot could not answer the customer’s question “How old are you?” (lines 18, 19) and sometimes took more than 5 s to reply (lines 4, 16, 18). In this example, the customers were ready to leave the shop when the robot suggested tasting a sample (line 31). As described above, in a user interface in which the buttons are arranged in various ways, there is the possibility that the opportunity to provide customer service may be lost depending on the operator and operation method. The user interface used in this experiment only required the operator to select an action according to a predetermined rule. Therefore, determining which button to press next and searching for that button were omitted, and as a result, the robot’s social presence was sufficient for the customers to accept its suggestions.

## 5. Verification of Effectiveness of the State-Transition Model for First Time Users

In this section, we investigate whether the robot can provide effective service for sales even if it is operated by users who have no previous experience in operating a robotic salesperson.

### 5.1. Experimental Environment

**Figure 20:**
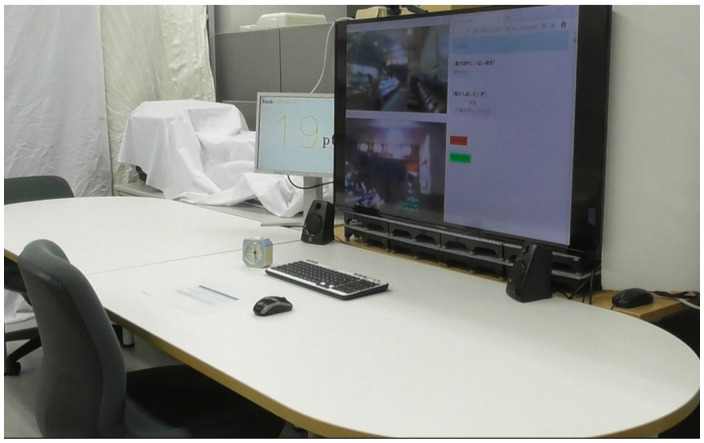
Experimental environment.

**Figure 21:**
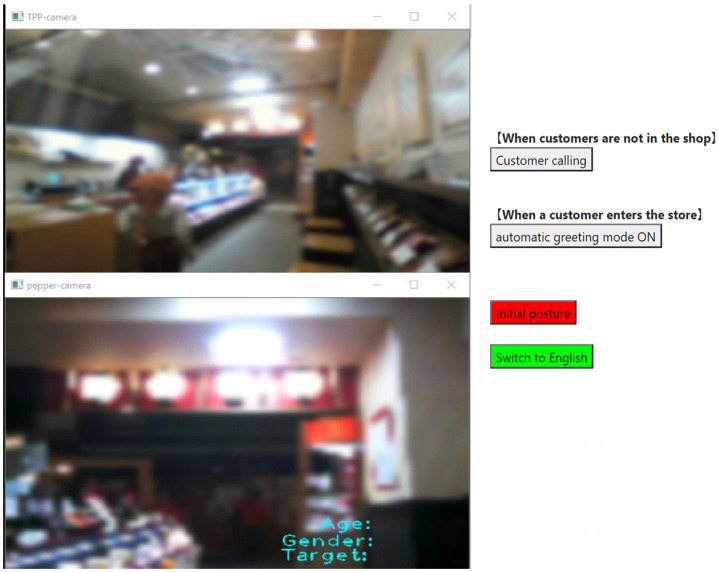
Operation screen.

Figure 20 displays the experimental environment in which participants remotely controlled the robot, and Figure 21 displays the robot operation screen presented to the participants. During the experiment, an experimenter observed the images from the camera placed behind the participant so that any problems could be addressed immediately. The settings at the shop in which the robotic salesperson was installed were the same as in Section 3 and Section 4.

### 5.2. Procedure

The participants listened to an explanation of the user interface for 10 min before the operation, and then operated the robot for 60 min. The explanation for both conditions specified that the task was to operate the robot installed in an actual shop in Kyoto to serve customers, and that the participants could operate the robot by judging the situation displayed on the screen based on the live video and clicking the appropriate buttons displayed on the screen with the mouse. After the operation, the participants were interviewed regarding their impression of the operation and the reasons for their actions during the experiment.

### 5.3. Participants

Eight participants (two females and six males) took part in the experiment. They were all 18–24-year-old university students living in Japan and were recruited for the experiment and paid for their contributions. None were known to the experimenters.

### 5.4. Evaluation Method

To investigate whether the robot could provide effective customer service even if it was operated by a user with no previous experience with the operation, we compared the ratio of customers who agreed to taste a sample in the three experimental conditions in this study: during operation by the participants using the user interface with the state-transition model, during operation by the experimenter using the user interface with the state-transition model, and during operation by the experimenter using the user interface without the state-transition model.

### 5.5. Results

In this experiment, 7 out of the 55 customer groups (12.7% of cases) agreed to the robot’s suggestion to taste a sample. To investigate whether the robot could properly provide service even when operated by participants who used the user interface for the first time, the customers’ tasting rate was compared with that in the experiment presented in Section 3 and Section 4. Here, the population parameter was the total number of customers with whom the robot interacted regardless of whether there was a conversation.

**Figure 22:**
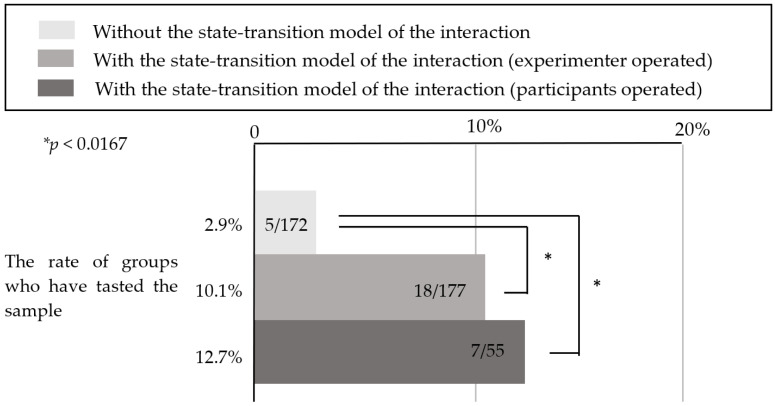
Ratio of customers who tasted a sample under each condition.

Fisher’s exact test revealed that there was a significant difference between these three groups (*p* < 0.05); therefore, multiple comparisons were performed between them. As there were three comparisons, the significance level was set to 0.0167 by Bonferroni correction. Figure 22 demonstrates that there were significant differences between the condition involving the state-transition model (operated by the experimenter) and the condition not involving the state-transition model, and between the condition involving the state-transition model condition (operated by the participants) and the condition not involving the state-transition model (*p* < 0.0167).

### 5.6. Discussion

From the results presented in Figure 22, the participants who had never operated a robotic salesperson could operate the robot as effectively as the experimenter. Therefore, we determined that the user interface based on the state-transition model of the interaction between the customers and robot developed in this study is suitable even for general users. During the post-experiment interview, many participants claimed that the operation was easy. This was because the user interface only required the operator to select a button according to the instructions presented on the screen based on the live video, and the robot could thus be easily operated even if the users did not have experience with the particular user interface. In addition, because the behavior of the robot was limited in the user interface used in this experiment and the experiment described in Section 4, there was no difference in the robot’s customer service even if the operation was performed by users with different levels of experience (i.e., experimenter and participants). 

During the operation, behaviors of the participants such as releasing the mouse, leaning against the chair, and looking away from the screen were observed when it was not necessary to give instructions to the robot (when visitors were not in the shop or when visitors were talking with the salespeople, etc.) Therefore, it is considered that the operators felt boredom when they did not need to give instructions to the robot. We have to consider how to motivate the operators while waiting for the next instructions.

During the post-experiment interview, participants provided various reasons why the operation was fun, such as “because I was able to operate the robot” and “because I was able to give instructions to the robot.” The reason for this was considered to be the novelty of remotely operating a robot installed in an actual shop in real time. Therefore, we have to think about how to keep the operators motivated after losing this novelty and enough time has passed. Opinions such as “I wish I had more freedom in operation” were obtained during the interview, and many participants mentioned the lack of freedom in choosing the control timing and behavior of the robot is the reason for why they did not feel a sense of accomplishment. Moreover, participants commented that they felt accomplished when the visitors responded to the robot’s handshake request or when the visitors entered the shop in response to the robot’s invitation. Thus, it shows that the operators felt fulfilled when their own choices and decisions made the robot successful in performing customer service. Therefore, it is necessary to investigate changes that can be made to the user interface according to these evaluations, such as changing the user interface to allow the operator to freely choose from several options, rather than our current user interface, in which one behavior of the robot is determined from one state of the customers. In this study, we compared robot operation based on the state-transition model and robot operation without the model; however, the dates of the experiments were far apart. Thus, it is possible that changes in the environment of the shop, such as changes in the product types and prices, may have also affected the results. These changes will have to be examined in future work.

## 6. General Discussion

In the field experiment, we performed a multimodal analysis and modeled the interaction between customers and the robotic salesperson as a state-transition model. Based on the model, we developed a user interface to easily operate the robot and demonstrated that it can sufficiently enhance the robot’s social presence for customers to accept its suggestions regardless of the user’s operational ability in a real-world environment. This was because the user interface only required the operator to select an action according to a predetermined rule. The operator thus did not have to determine the button to be pressed next and search for that button on the screen. Therefore, creating a state-transition model from the interaction simplifies crowdsourced robot control for the general user to enhance the robot’s social presence for efficient customer service. In this study, we focused only on simplifying the operation. However, in order for the general user to keep operating, in addition to the operation’s simplicity, they need an incentive to be entertained by the operation similar to a game. Investigation of such incentives will be explored in the future. According to a previous research, it is important for a human to accept the role and usefulness of robots to be able to collaborate and work with them efficiently. Human perception of robots is not only a technological issue, but also a cultural one [27]. As we conducted our study in Japan in a specific type of shop during a specific time period, investigating this approach in other cultures and for different time spans can yield interesting results in the future. In addition, we believe that these users performed operations may be replaced by machine learning in future works.

## 7. Conclusions

In this study, our goal was to develop a user interface for general users, allowing them to remotely control a robotic salesperson installed in a real-world environment and enhance its social presence for customers to accept its suggestions. We investigated the typical flow of the customer–robot interaction that was effective for sales promotion and created a state-transition model with automatic functions by accessing the robot’s sensor information. Thereafter, we examined whether the model was effective in an actual shop when the robot was operated by an experimenter using the user interface based on the model. Furthermore, we conducted experiments to test whether first-time users could operate the robot properly and enhance its social presence. The results revealed that the user interface with this model allowed the operator to lead more customers to agree to the robot’s suggestion to taste a sample even if the operator was a first-time user. The state-transition model allowed its users to simply follow instructions displayed on the screen of the user interface, while some of the robot’s behavior was automated by accessing real-time sensor information, which simplified the operation. Therefore, creating a state-transition model from the interaction can allow crowdsourced robot control to become an easier experience for the general user to facilitate efficient customer service.

## Figures and Tables

**Table 1:** Optimized parameters of weights obtained from the training.

Cues.	Weights
X1 (Eye Contact)	0.53
X2(Duration of Eye Contact)	1.35
X3 (Distance)	−0.99
X4 (Approaching)	0.17
X5 (Laughing)	1.20

**Table 2:** Conversation in baseline condition (20 September 2018).

1	C1	((Entering the Shop))
2	C1	((looking at the products))
3	P	Hey! (0.6)
4	C1	((looking at the products and moving towards Pepper)) (1.0)
5	P	Hello!
6	C1	((looking at the products)) (2.0)
7		((Pepper was ignored))

P = robot, C1 = woman.

**Table 3:** Conversation in the attention estimation model condition (10 September 2018).

1	C1	((Entering the Shop))
2	C1	((looking at the products and approaching the robot))
3	P	Hey! (1.0) Hey!
4	C1	((turned her head and looked at the robot)) (0.6)
5	P	Hello!
6	C1	Hello↑!

P = robot, C1 = woman.

**Table 4:** Conversation with a group of customers who looked at the sample and agreed to taste it after the robot’s suggestion with the assistance of a salesperson (5 December 2017).

1	C1, C2	((Entering the Shop))
2	C1	((Approaching the robot with her camera))
3	P	Hello. =
4	C1	= [Hi!.
5	C2	[Hello. (2.8)
6	P	My name is Pepper.=
7	C1	= Hi, Pepp [er!
8	P	[Nice to meet you. =
9	C2	= Nice to meet you too. =
10	C1	= Nice to meet you too. (1.0)
11	C1	Hi, Pepper::
12	P	May I shake hands with you?
13	C1	Sure! Hi! Hello↑. ((Shake hands with the robot))
14	P	Nice to meet you. =
15	C1	= Nice to meet you. ((touching the robot’s hand))
16	P	Would you like to try a sample? You can taste here.
17	C1, C2	((looking at the samples))
18	C1	OK! (). (7.0)
19	P	Excuse me. =
20	C1	= Hi. ((looking at the robot)) =
21	P	= Please give a sample.
22	S	((coming to give the sample to them))
23	C1, C2	((looking at S))
24	C1	Sample?
25	S	((giving C1 the sample))
26	C1	((trying to taste))
		(…)
27	C1	((purchasing the product))

P = robot, C1 = woman, C2 = woman, S = salesperson.

**Table 5:** Conversation with a group that looked at the sample but did not taste it after the robot’s suggestion (27 November 2017).

1	C1, C2	((Looking at the Robot From Outside of the Shop))
2	P	May I shake hands with you?
3	C1	((approaching the robot and shaking hands with it)) (1.8)
4	P	Thank you.
5	C1	Oh ((being surprised))
6	C1	((turning away from the robot)) hhh.
7	C2	hh.
8	P	Would you like to try a sample?
9	C1	((Turning to the robot))
10	P	You can taste here (1.6)
11	C1	((Looking at the sample))
12	C2	That’s amazing. How can you ().((looking at the robot))
13	C1	((Looking away from the sample while nodding))
14	P	You can taste very hot, medium hot and yuzu shichimi. (2.1)
15	C2	Thank you. ((Looking away from the robot))
16	C1	No, thank you.
17	C2	hh. (3.2)
18	P	Excuse me. Please give them a sample.
19	C1, C2	((Turning away from the robot))

P = robot, C1 = woman, C2 = man.

**Table 6:** Conversation with a group that did not look at the sample or taste it after the robot’s offer (5 December 2017).

1		((Entering the shop))
2	C1	((Approaching the robot))
3	C1	((Waving his hand in front of the robot’s face))
4	P	Hello.
5	C1	[oh], hello:.
6	C2	[hello:]. (0.8)
7	C2	he↑llo:. (1.0)
8	P	My name is Pepper.=
9	C2	=ok! (0.5)
10	P	Nice to meet you.
11	C2	Nice to meet you, too:.
12	C1	Pepper, sing someth[ing:.]
13	P	[May] I shake hands with [you?
14	C1	Ah ok. ((shaking hands with the robot)) thanks. (3.4)
15	C2	((Looking away from the robot))
16	C1	Pepper, [sing:
17	P	[Nice] to meet you. (3.3)
18	C1	((Waving his hand in front of the robot’s face))
19	C1	((Looking away from the robot)) (2.8)
20	P	Would you like to try a sample?=
21	C1	=Wow! That’s amazing!=
22	P	=You can taste here.
23	C1, C2	((Leaving without looking at the sample))

P = robot, C1 = man, C2 = woman.

**Table 7:** Behavior of customers when the robot asked for the salesperson’s assistance.

		before the Robot’s Request from the Salesperson	
		Groups Who Looked at the Sample	Groups Who did not Look at the Sample
After the Robot’s Request from the Salesperson	Groups who tasted the sample	7	1
	Groups who did not taste the sample	0	8

**Table 8:** Responses when the robot offered a sample.

		before the Robot’s Suggestion	
		Groups who Looked at the Robot	Groups Who did not Look at the Robot
After the robot’s suggestion	Groups who looked at the robot	39	8
	Groups who did not look at the robot	3	46

**Table 9:** Conversation between a group that tasted a sample and the robot with the state-transition model (21 November 2019).

1	C1	Hello, Everyone.
2	P	Hey. Hello. (0.5)
3	P	May I shake hands with you?
4	C2	Speak English!
5	C1	Yeah, Yeah. You can
6	C2	Yes! ((shaking hands with the robot))
7	P	My name is Pepper. Nice to meet you.
8	C2	Nice to meet you too. (4.0)
9	P	Would you like to try a sample? You can taste here.
10	C1, C2	((Looking at the sample))
11	C1	You can try medium hot, very hot, yuzu shichimi. Please find your favorite spiciness! (3.0)
12	C1	((tasting))

P = robot, C1 = man, C2 = woman.

**Table 10:** Conversation in which the robot could not continue the conversation without the state-transition model (17 April 2019).

1	P	Hey. ((C1,C2 Look at the Robot))
2	C1	Huhu[hu.
3	P	[Hello.
4	C1	Hi!=
5	C2	Hello! (2.4)
6	P	My name is Pepper.(1.8)
7	C2	How do you do? (2.0) ((Looking at the sample))
8	P	Nice to meet you.
9	P	I’m good. (2.9)
10	P	How are you? (.)
11	C1	((Looking at the robot)) I’m fine. You too?
12	P	Oh, that’s nice! (2.1)
13	C1	((Looking away from P))
14	P	Can I sing an original rap about Shichimi?
15	C1, C2	((Leaving the shop))

P = robot, C1 = man, C2 = woman.

**Table 11:** Conversation with a group that ignored the robot’s suggestion to taste a sample without the state-transition model (17April 2019).

1	C1, C2	((Entering the Shop))
2		(4.8)
3	C1	Hello! (1.0)
4	P	Hey. Hello.
5	C1	Hi! (1.1)
6	C1	How are you? (5.2)
7	P	I’m good. (.)
8	C2	Oh! [hh.
9	C1	[hhh.(3.5)
10	P	My name is Pepper (1.2)
11	C1	[Ah:::.
12	C3	[Ah: (.)
13	P	Nice to meet you.
14	C3	Nice to meet you↑.=
15	C2	=Nice to meet you. (3.8)
16	C1	Hello? (holding the camera) (.)
17	P	May I shake hands with you? (1.7)
18	C1	Yes. Yes! ((shaking hands with the robot))
19	P	Thank you! (.)
20	C1	Thank you↑. (2.0)
21	C1	((Looking back and looking away from the robot))
22	C1	((leaving the shop)) (4.5)
23	P	Would you like to try a sample?
24	C2	((Looking back and leaving the shop))
25	P	You can taste here.

P = robot, C1 = man, C2 = man.

**Table 12:** Conversation with a group whose behavior did not match the model (21 November 2019).

1	C1, C2	((Entering the shop))
2		(10.4)
3	C1	Hello! (1.2)
4	P	Hey. Hello.
5	C2	What’s your [name?
6	P	[May I shake hands with you?
7	C1	(.) Yes! ((shaking hands with the robot)) (2.0)
8	P	My name is Pepper. (.)
9	C1	Pepper? (.)
10	P	Nice to meet you. (1.2)
11	C1	(?)((Requesting a handshake from the robot again))
12	P	Touch tablet and select. Can I record our behavior?
13	C1	((Touching the tablet))
14	P	Would you like to try a sample? You can taste here.
15	P	If you like spicy foods, why don’t you try very hot Shichimi?(2.2)
16	C1	I don’t like that food. But I like you! (1.1)
17	P	You can try medium hot, very hot and yuzu Shichimi. Please find your favorite spiciness.(1.9)
18	C1	No. Can I (?) ((Requesting a handshake)) (3.2)
19	P	Do you know Shichimi?
20	C1	((Leaving the shop))

P = robot, C1 = man, C2 = man.

**Table 13:** Conversation in which it took a long time to select a button without the state-transition model (17 April 2019).

1	C1	Hello! (3.1)
2	C1	((Looking at the robot’s face)) (4.4)
3	P	Hello. (1.8)
4	C1	Ohayo gozaima:su (6.2)
5	C1	Konnichiwa. (.)
6	P	My name is Pepper.=
7	C2	You speak English? (.)
8	C1	You speak [English?
9	C1	[konni]chiwa.
10	C1	hhh.
11	C2	What’s your name? (3.2)
12	P	Hello. My name is Pepper.(.)
13	C2	Pepper.=
14	C1	=Where are you [from?]
15	P	[Nice] to meet you. (.)
16	C1	Where you come from? (6.9)
17	P	I’m from Japan.
18	C1	Ah (?) Japan. (?) How old are you? (10.4)
19	P	Sorry. Could you say again?
20	C3	How old are you? (2.0)
21	P	I don’t know.
22	C3	No. hhhh ((C1,C2,C3 turning away from the robot))
23	C1	((Looking back at the robot)) You are so [(?).]
24	P	[May] I shake hands with you? (.)
25	C1	Sorry. (4.3)
26	P	May I shake hands with you? (.)
27	C1	Oh. ((looking back at C2,C3))
28	C3	((Shaking hands with the robot)) (3.0)
28	P	Nice to meet you.
29	C1	OK! Byebye!((C1,C2,C3 are trying to leave the shop))
30	P	Would you like to try a sample? You can taste here.
31	C1	We have to go::.((waving to the robot and leaving the shop))

P = robot, C1 = woman, C2 = woman, C3 = man.
